# Molecular Aspects of Geriatric Pharmacotherapy

**DOI:** 10.3390/cells14171363

**Published:** 2025-09-01

**Authors:** Patryk Rzeczycki, Oliwia Pęciak, Martyna Plust, Marek Droździk

**Affiliations:** Department of Experimental and Clinical Pharmacology, Pomeranian Medical University, 72 Powstańców Wielkopolskich Avenue, 70-111 Szczecin, Poland; patryk.rzeczycki@pum.edu.pl (P.R.); peciak.oliwia@gmail.com (O.P.); mplust16@gmail.com (M.P.)

**Keywords:** geriatric, molecular aspects, pharmacology, drug interactions

## Abstract

Pharmacotherapy in the geriatric population is one of the greatest challenges in modern medicine. Elderly patients, characterized by multimorbidity and the resulting polypharmacy, are significantly more exposed to adverse drug reactions (ADRs), which often lead to hospitalization and a decline in quality of life. Understanding the reasons for this difference requires an analysis of the physiological changes that occur during the aging process at the molecular level. This article presents a perspective on the molecular aspects of geriatric pharmacotherapy, focusing on the fundamental mechanisms that are modified with age. The analysis covers changes in pharmacokinetics, including the role and regulation of cytochrome P450 (CYP) enzymes, whose activity, especially in phase I reactions, is significantly reduced. The age-dependent dysfunction of drug transporters from the ABC (ATP-binding cassette) and SLC (solute carrier) families in key organs such as the intestines, liver and kidneys is discussed, which affects the absorption, distribution and elimination of xenobiotic compounds, including drugs. The article also provides a comprehensive analysis of the blood–brain barrier (BBB), describing changes in neurovascular integrity, including the dysfunction of tight junctions and a decrease in the activity of P-glycoprotein, sometimes referred to as multidrug resistance protein (MDR). This increases the susceptibility of the central nervous system to the penetration and action of drugs. In the realm of pharmacodynamics, changes in the density and sensitivity of key receptors (serotonergic, dopaminergic, adrenergic) are described based on neuroimaging data, explaining the molecular basis for increased sensitivity to certain drug classes, such as anticholinergics. The paper also explores new research perspectives, such as the role of the gut microbiome in modulating pharmacokinetics by influencing gene expression and the importance of pharmacoepigenetics, which dynamically regulates drug response throughout life via changes in DNA methylation and histone modifications. The clinical implications of these molecular changes are also discussed, emphasizing the potential of personalized medicine, including pharmacogenomics, in optimizing therapy and minimizing the risk of adverse reactions. Such an integrated approach, incorporating data from multiple fields (genomics, epigenomics, microbiomics) combined with a comprehensive geriatric assessment, appears to be the future of safe and effective pharmacotherapy in the aging population.

## 1. Introduction

The aging of the population is one of the most significant social aspects of the 21st century. With increasing life expectancy, the proportion of elderly people, who are the largest group of medication users, is growing. This demographic shift presents healthcare systems with fundamental challenges, one of the most important being the provision of safe and effective pharmacotherapy. Geriatric patients are not just “older adults”; their bodies undergo complex, multi-level physiological (involutive) changes that fundamentally alter their response to drugs. Ignoring these differences leads to serious clinical consequences, including more frequent adverse drug reactions (ADRs), higher incidence of major geriatric problems that often results in hospitalization and increased mortality [1,2,3,4,5].

**Figure 1:**
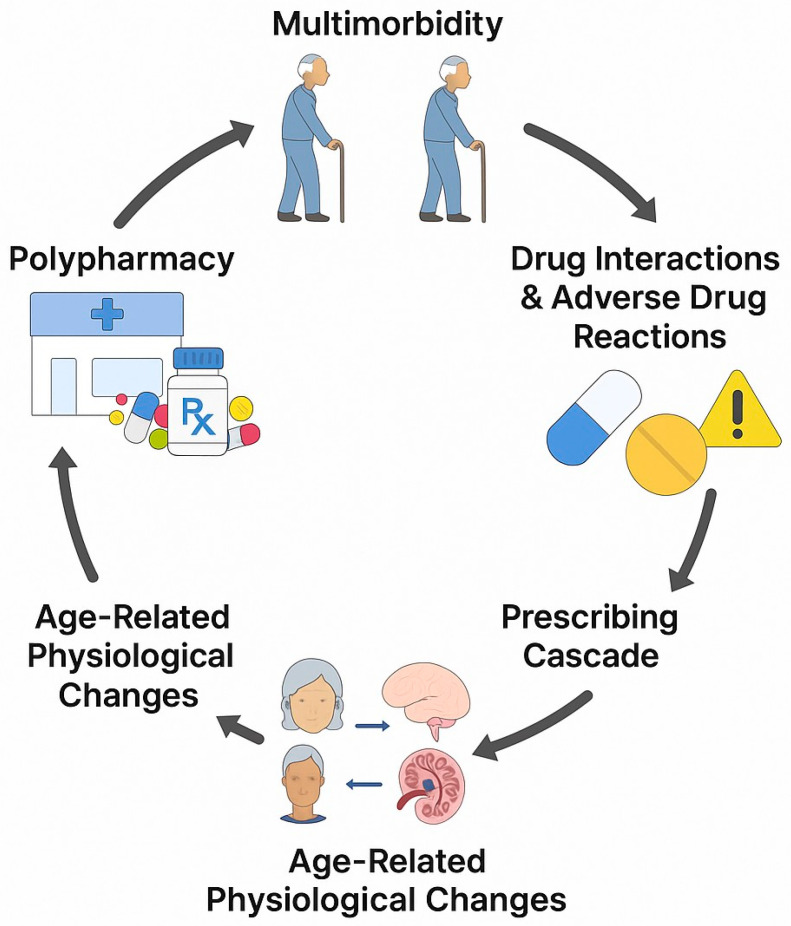
Circle of chronic pharmacotherapy in the elderly. Created in Canva, Krita and other free graphic programs.

A clinical reflection of these therapeutic problems is the phenomenon of polypharmacy. It is most often defined as the simultaneous use of five or more drugs, which affects more than half of the population over 65 in Poland [6]. Polypharmacy is inextricably linked with multimorbidity, i.e., the coexistence of multiple chronic diseases, which is characteristic of old age. However, the number of preparations taken is not the only source of the problem. A key, often underestimated mechanism, is the “prescribing cascade”. It consists of misinterpreting an ADR as a new disease symptom, which leads to prescribing another preparation to treat it [7,8]. This process creates a vicious cycle that drives polypharmacy and increases the risk of further interactions and ADRs (Figure 1).

An example is the induction of parkinsonian symptoms by antipsychotic drugs, such as haloperidol (one of the pioneering antipsychotics). In a geriatric patient, where motor slowing and tremor can be mistakenly considered the onset of idiopathic Parkinson’s disease, this can result in the often unnecessary introduction of drugs modulating the dopaminergic system. One clinical clue could be the assessment of head tremor, which should not be present in Parkinson’s disease [9,10,11]. This is an example of the relationship between clinical pharmacology and other fields of medicine. Improperly used drugs, especially when interacting, carry the risk of their own adverse effects, such as orthostatic hypotension or delirium, which further complicates the clinical picture and significantly hinders the treatment regimen.

The driving force for this cascade are fundamental, age-related changes in pharmacokinetics and pharmacodynamics, which significantly increase the likelihood of the primary ADR occurring. The altered response of the body to the drug means that standard doses become too high, and adverse effects that are rare in younger patients become common in the geriatric population. Therefore, drug doses should necessarily be adjusted to the patient’s age and the function of organs such as the liver or kidneys [12,13,14].

To fully understand and effectively manage geriatric pharmacotherapy, it is necessary to move beyond a simple categorization of physiological changes towards a deeper approach. The “geroscience” paradigm offers such a perspective [15]. It assumes that the same, common cellular and molecular mechanisms underlie most age-related diseases and the biological aging process itself. Aging is not seen as a sum of independent defects, but as a result of the accumulation of damage at a fundamental level, such as genomic instability, DNA damage by free radicals, mitochondrial dysfunction, loss of proteostasis (protein homeostasis), cellular senescence, telomere shortening, increased oxidative stress, etc. [16,17,18,19,20,21,22]. According to this view, the changes in pharmacokinetics and pharmacodynamics observed in the elderly are not random events, but consequences of these basic aging processes. This is further proof of the immense significance of involutive changes in the context of pharmacotherapy. The decrease in the activity of metabolic enzymes, the weakening of drug-transporter function or the increased permeability of the blood–brain barrier are manifestations of deeper, molecular dysfunctions [23,24,25]. This perspective may have revolutionary therapeutic implications. It suggests that in the future, pharmacotherapy may shift its goal from treating single, symptomatic diseases of old age to targeting the mechanisms of aging themselves. Numerous studies in mice prove this [26,27].

Recent advances highlight two emerging strategies to improve pharmacotherapy outcomes in older adults: pharmacoepigenetics and microbiota-directed interventions.

Other established approaches to individualizing pharmacotherapy in this population include pharmacogenetic testing and therapeutic drug monitoring. Pharmacogenetics enables the identification of genetic variants that influence drug metabolism and response, while therapeutic drug monitoring allows for dose adjustment based on measured plasma drug concentrations, thereby improving safety and efficacy [18]. Although these strategies have proven clinical value, their application is often limited to specific drugs or therapeutic classes. In contrast, pharmacoepigenetics and microbiota-directed interventions have the potential to modulate fundamental biological processes that affect multiple drug pathways simultaneously, offering a broader and potentially more transformative impact on geriatric pharmacotherapy.

Pharmacoepigenetics examines how age-related epigenetic modifications influence drug metabolism and response, potentially enabling more precise and safer prescribing. Microbiota modulation—through dietary changes, probiotics or prebiotics—has been shown to affect drug bioavailability, reduce adverse effects and enhance therapeutic efficacy. These approaches, discussed in detail later in this paper, may complement established strategies for minimizing polypharmacy and its associated risks. In addition to pharmacological approaches, non-pharmacological geroprotective interventions are essential to slow biological ageing and preserve functional capacity. Agents such as rapamycin have demonstrated the potential to extend lifespan and improve health markers in animal models [26,27], while regular physical activity and balanced nutrition remain the most effective measures to reduce frailty and chronic disease burden [18].

To actively disrupt the vicious cycle of chronic pharmacotherapy, targeted clinical interventions can be applied. These include regular medication reviews, early identification and management of adverse drug reactions, deprescribing unnecessary medications, and personalized programmes incorporating physical activity and dietary optimization. When implemented systematically, such measures can decrease polypharmacy, enhance functional status and improve quality of life in older adults [18,26,27].

## 2. Pharmacokinetic Changes at the Molecular Level

Pharmacokinetics, describing the fate of a drug in the body through the processes of liberation, absorption, distribution, metabolism and excretion (LADME), undergoes profound and multifaceted modifications during the aging process. These changes are not just a simple slowing of function, but complex, often opposing modifications at the cellular and molecular level that determine the drug concentration at the site of action, its duration of action and the risk of toxicity. Understanding these mechanisms is the foundation of rational geriatric pharmacotherapy [19,20,21,22].

### 2.1. Absorption

Although general changes in drug absorption from the gastrointestinal tract are often considered clinically insignificant for most substances [22], there are specific molecular mechanisms whose modification with age is of great importance.

Changes in pH and motility: One of the key changes is an increase in gastric pH, resulting from reduced secretion of hydrochloric acid, which leads to atrophic gastritis or the widespread use of proton pump inhibitors (PPIs). Higher pH impairs the absorption of drugs requiring an acidic environment for optimal absorption, such as calcium carbonate or some antifungal drugs (e.g., itraconazole). Furthermore, elevated gastric pH can lead to the premature breakdown of enteric-coated drugs (e.g., acetylsalicylic acid), increasing the risk of gastrointestinal irritation and the development of erosions, which can lead to gastrointestinal bleeding [23]. Simultaneously, a slowing of gastrointestinal motility is observed, including delayed gastric emptying. This can delay the drug’s arrival in the small intestine, the main site of absorption, which in turn delays the onset of action and reduces the peak concentration of drugs absorbed in the proximal intestine, such as paracetamol [24].

Active transport: Another important mechanism is the decrease in the efficiency of active (ATP-dependent) transmembrane transport. With age, the number of intestinal villi decreases, which reduces the total absorption surface area. More importantly, the efficiency of transport systems responsible for the absorption of key nutrients and vitamins, such as B vitamins (B1, B6, B12), folic acid, calcium or iron, decreases. This molecular deficit can contribute to nutritional deficiencies, often observed in the geriatric population, and may require supplementation in higher doses or parenteral administration. A deficiency of B vitamins can lead to the development of progressive polyneuropathy, the consequences of which can be very serious, as they lead to dysfunction in many systems [25].

### 2.2. Distribution: Changes in Body Composition and Protein Binding

The distribution process, i.e., the dispersion of a drug throughout the body, is determined by body composition, tissue blood flow (vascularization of organs and tissues) and the degree of binding to plasma proteins. All these factors undergo significant changes with age [26].

Lipophilicity versus hydrophilicity: A fundamental change is the modification of body composition: the percentage of body fat increases, even with a stable or decreasing body weight, while the total body water content decreases. This dichotomy has opposing effects on different groups of drugs.

Lipophilic drugs (fat-soluble): The increased amount of adipose tissue leads to an increase in the volume of distribution (Vd) for lipophilic drugs, such as benzodiazepines (e.g., diazepam), antipsychotics (e.g., chlorpromazine) or some cardiac drugs (e.g., amiodarone). A larger Vd means the drug is more widely distributed and stored in fatty tissue. This results in a lower initial blood concentration and, at the same time, a significant extension of its half-life (t1/2), which can be very long to begin with [28]. With regular dosing, this leads to the accumulation of the drug and its metabolites, increasing the risk of toxicity and delaying the resolution of its effects after discontinuation.

Hydrophilic drugs (water-soluble): The reduced water content in the body causes a decrease in the volume of distribution for hydrophilic drugs. This group includes, among others, digoxin, morphine, theophylline, aminoglycoside antibiotics or some diuretics (e.g., hydrochlorothiazide) [29]. A smaller Vd means that after a standard dose, the drug reaches a higher concentration in plasma and tissues, which increases the risk of reaching a toxic level early in therapy [30].

Plasma protein binding: The degree of drug binding to plasma proteins determines its pharmacologically active, free fraction. With age, there are changes in the concentration of two key transport proteins:

Albumin: The concentration of albumin, the main protein that binds drugs, tends to decrease, especially in malnourished patients or those with chronic diseases. A 15–20% decrease in albumin concentration compared to younger people is quite common. This leads to an increase in the free fraction of drugs that are highly bound to albumin (>90%), such as warfarin, digoxin or non-steroidal anti-inflammatory drugs (NSAIDs) [31]. Even a small increase in the free fraction (e.g., from 1% to 2%) means a doubling of the amount of active drug, which can dramatically intensify its effect and toxicity. This clinical aspect must be absolutely considered in pharmacotherapy [32].

α1-acid glycoprotein (AAG): The concentration of this protein, which is an acute-phase reactant, often increases with age, especially during inflammatory conditions and chronic diseases. AAG mainly binds basic drugs, such as propranolol, amitriptyline or mianserin. An increase in AAG concentration can lead to increased binding of these drugs and a decrease in their free, active fraction, which could theoretically weaken their effect [33].

These opposing changes in transport protein concentrations further complicate the prediction of clinical effects and highlight the importance of individual patient assessment.

### 2.3. Hepatic Drug Metabolism

The liver is the central organ of drug metabolism, and its function undergoes significant modifications in the aging process. These changes affect the organ’s blood flow, its mass and, most importantly, the activity of key enzyme systems [34].

Molecular basis of drug metabolism: Drug metabolism in the liver (biotransformation) occurs in two main phases.

Phase I reactions mainly include oxidation, reduction and hydrolysis. Their purpose is to introduce or unmask functional groups in the drug molecule, which usually leads to its inactivation, but sometimes to its activation (in the case of prodrugs like clopidogrel). The key role in these reactions is played by the cytochrome P450 (CYP) group of proteins, located mainly in the smooth endoplasmic reticulum of hepatocytes [35]. Phase II reactions (conjugation) involve attaching an endogenous, polar molecule (e.g., glucuronic acid, sulfate, glutathione) to the drug or its phase I metabolite, which significantly increases its water solubility and facilitates its excretion from the body [36].

Changes in phase I and phase II in aging: One of the fundamental principles of geriatric pharmacotherapy is the observation that the aging process affects both phases of metabolism differently.

Decrease in phase I reaction efficiency: With age, a significant impairment of phase I reactions is observed, especially those catalyzed by CYP enzymes. This decrease is the result of several overlapping factors: a reduction in the active liver parenchyma mass (by up to 20–40%), a reduction in hepatic blood flow (by 20–50%) and a decrease in the activity of the microsomal enzymes themselves. It is estimated that after the age of 40, the overall activity of CYP450 can decrease by about 30%, and the clearance of drugs metabolized by this route can be reduced by 30–40% [37]. This leads to a prolonged half-life and an increased risk of accumulation for many drugs, including benzodiazepines, tricyclic antidepressants, some antiarrhythmic drugs and calcium channel blockers [38].

Relative preservation of phase II reactions: In contrast to phase I, conjugation reactions (phase II) are generally well preserved in healthy older adults. This observation has key clinical significance—when choosing drugs for geriatric patients, those metabolized mainly by conjugation rather than oxidation should be preferred (e.g., in the benzodiazepine group, lorazepam and oxazepam are safer than diazepam) [39]. However, it should be noted that in patients with frailty syndrome and severe inflammation, phase II metabolism may also be impaired [40].

Changes in CYP isoforms and first-pass effect: The CYP450 family exists as many isoforms, and their activity changes with age to varying degrees. The most clinically significant are changes in the activity of CYP3A4, CYP2D6, CYP2C19 and CYP1A2, which are responsible for the metabolism of most commonly used drugs. A decrease in their activity is one of the main risk factors for ADRs [41]. Particularly important is the effect of aging on the first-pass effect, which is the metabolism of a drug in the liver (and intestinal wall) after absorption from the gastrointestinal tract and before it reaches systemic circulation. The efficiency of this process decreases by about 1% per year after the age of 40. As a result, the oral bioavailability of drugs with a high hepatic extraction ratio (e.g., propranolol, verapamil, nifedipine, nitrates) increases significantly in older people, which with standard dosing leads to higher plasma concentrations and intensified effects [42].

Regulation and genetic polymorphism: Drug response is further modified by genetic and environmental factors. The expression and activity of CYP enzymes are regulated by hormones (e.g., testosterone deficiency in old age reduces CYP activity), inflammatory states (pro-inflammatory cytokines, such as IL-6 and TNF-α, inhibit the activity of many isoforms) and diet (e.g., grapefruit juice inhibits CYP3A4) [43,44,45]. Age-related changes in the activity of key CYP isoforms and their clinical consequences are summarized in Table 1. Moreover, the genes encoding CYP enzymes are highly polymorphic, which leads to the existence of different metabolic phenotypes in the population: Ultra-rapid metabolizers (UM): Have duplicated or more active alleles of the gene. Extensive (normal) metabolizers (EM): Have two functional alleles. Intermediate metabolizers (IM): Have one allele with reduced function. Poor metabolizers (PM): Have two non-functional alleles [46]. In a geriatric patient who is also a poor metabolizer for a given pathway (e.g., CYP2D6), the age-dependent decrease in liver function overlaps with the genetically determined deficit, which drastically increases the risk of toxicity of drugs metabolized by this route [47]. It is also worth noting that the ability for enzymatic induction by some drugs (e.g., rifampicin, carbamazepine) may be weakened in the elderly. This means that drug interactions involving the acceleration of another drug’s metabolism may be less pronounced and more difficult to predict [48].

### 2.4. Drug Transporters: Changes During the Aging

Hepatic metabolism is only one of the elements determining the fate of a drug in the body. An equally crucial, though formerly less appreciated role, is played by transport proteins, which, like molecular gates, control the flow of xenobiotics across biological barriers. Their function is essential for the processes of absorption, distribution and elimination of drugs, and their age-related dysfunction is of fundamental clinical importance [54,55].

ABC and SLC superfamilies: Transport proteins can be divided into two main superfamilies: ABC (ATP-Binding Cassette) Transporters: This is a family of about 50 proteins that act as active efflux pumps. They use energy from ATP hydrolysis to transport substrates against their concentration gradient, usually out of the cell or into the lumen of canaliculi (e.g., bile, renal). They perform a key protective function, limiting the body’s exposure to toxins and drugs [56,57]. And SLC (solute carrier) Transporters: This is a much larger and more diverse family, with over 400 proteins. They mainly operate through facilitated or active transport, moving substrates along an electrochemical gradient or using an ion gradient (e.g., Na+ or H+). They are responsible for both the uptake (influx) of drugs and endogenous substances into cells (e.g., in hepatocytes, renal tubule cells) and their efflux [58,59]. The aging process affects the expression and function of many of these transporters, which has a direct impact on pharmacokinetics.

P-glycoprotein (P-gp/ABCB1): This is the best-known and clinically most important transporter from the ABC family. P-gp is located in the apical membrane of epithelial cells in key barriers: in the intestine (limits drug absorption), in the bile canaliculi of hepatocytes (responsible for secretion into bile), in the proximal tubules of the kidneys (secretion into urine) and on the endothelial cells of the blood–brain barrier (active efflux of drugs from the brain). Studies indicate a general decrease in P-gp expression and function with age. For example, a PET study demonstrated significantly reduced P-gp activity at the human blood–brain barrier in elderly subjects compared to younger adults [60] This decrease in the intestine can paradoxically increase the bioavailability of some drugs, but reductions in the liver, kidneys and brain impair elimination and elevate tissue exposure to drugs and toxins [61,62].

BCRP (Breast Cancer Resistance Protein/ABCG2): Another important efflux transporter from the ABC family, with broad substrate specificity, including statins and anticancer drugs. Its expression may also change with age, affecting the pharmacokinetics of its substrates [63,64].

Organic Anion and Cation Transporters (OATs/OCTs/OATPs): These proteins from the SLC family are crucial for the uptake of drugs from the blood into hepatocytes and renal tubule cells, where they initiate the process of active secretion [65,66]. Studies on these transporters show that their expression increases sharply after birth and peaks in adulthood, after which it declines during the aging process [67]. The decline in OAT and OCT function in the kidneys is, besides the decrease in GFR, a major cause of impaired excretion of many anionic and cationic drugs [68].

A particularly interesting phenomenon that may explain the mechanism underlying these changes is that the decline in transporter function in the aging organism may be a post-transcriptional event. Studies on aged mouse models have shown that while mRNA levels for ABC transporters in brain microvessels remain unchanged, protein levels are significantly reduced [69]. This suggests that the decline stems not from reduced transcription, but from post-transcriptional processes such as protein folding, trafficking or stabilization in the cell membrane. This dysfunction at the level of protein homeostasis (proteostasis) is one of the fundamental, well-documented hallmarks of aging [70,71]. An integrated view of the role of transporters is offered by the “remote sensing and signaling hypothesis”. According to it, transporters in the gut–liver–kidney axis do not operate in isolation but form a coordinated communication network between organs. They regulate not only the fate of xenobiotics but also the fate of endogenous metabolites, signaling molecules and products produced by the microbiome [72,73,74].

### 2.5. Renal Excretion

The kidneys play a key role in the elimination of many drugs and their metabolites, and their function inevitably deteriorates with age. It is said that this pathway undergoes the greatest involution in function during aging [75]. Decline in GFR and Tubular Function: After the age of 40, the glomerular filtration rate (GFR) decreases by an average of 8–10 mL/min/1.73 m^2^ per decade [76]. This means that in an 80-year-old person, the GFR can be 30–50% lower than in a young adult. Concurrently, renal blood flow and tubular function, including secretion and reabsorption, also decline [77]. This renal impairment leads to prolonged half-life and reduced clearance of drugs eliminated renally, requiring careful dose adjustment. Drugs affected include ACE inhibitors, digoxin, metformin, aminoglycosides and NSAIDs. This decline in renal function leads to a prolonged half-life and reduced clearance of drugs eliminated by this route, requiring careful dose modification. Drugs whose dosage must be adjusted to renal function include ACE inhibitors (ACEIs), digoxin, metformin, aminoglycoside antibiotics and non-steroidal anti-inflammatory drugs [78,79].

Role of Renal Transporters: It should be emphasized that reduced renal excretion is not solely the result of a decrease in filtration. It is also a direct consequence of impaired active (ATP-dependent) tubular secretion, a process entirely dependent on drug transporters. As mentioned earlier, the age-related decrease in the expression and function of OAT and OCT transporters in the basolateral membrane and MRP and MATE transporters in the apical membrane of proximal tubule cells directly limits the kidneys’ ability to actively remove drugs from the blood into the urine. Thus, even with a seemingly normal GFR, the ability to eliminate certain drugs may already be significantly impaired (see Table 2) [80,81,82]. Clinical Assessment of Renal Function: A key problem in clinical practice is the proper assessment of renal function in the elderly. Serum creatinine concentration, a commonly used indicator, is highly misleading in this population. Due to age-related muscle mass atrophy (sarcopenia), creatinine production is reduced. As a result, its serum concentration may remain within the normal laboratory range, even with a significantly reduced GFR. Therefore, in every elderly patient, GFR should be estimated using appropriate formulas, such as the Cockcroft–Gault formula or the MDRD (Modification of Diet in Renal Disease) and CKD-EPI (Chronic Kidney Disease Epidemiology Collaboration) formulas. The Cockcroft–Gault formula, despite its limitations, is still often recommended because most recommendations for drug dosing in renal failure were historically developed based on this very formula [83,84,85,86].

## 3. Biological Barriers in the Aging Organism: The Blood–Brain Barrier (BBB)

**Figure 2:**
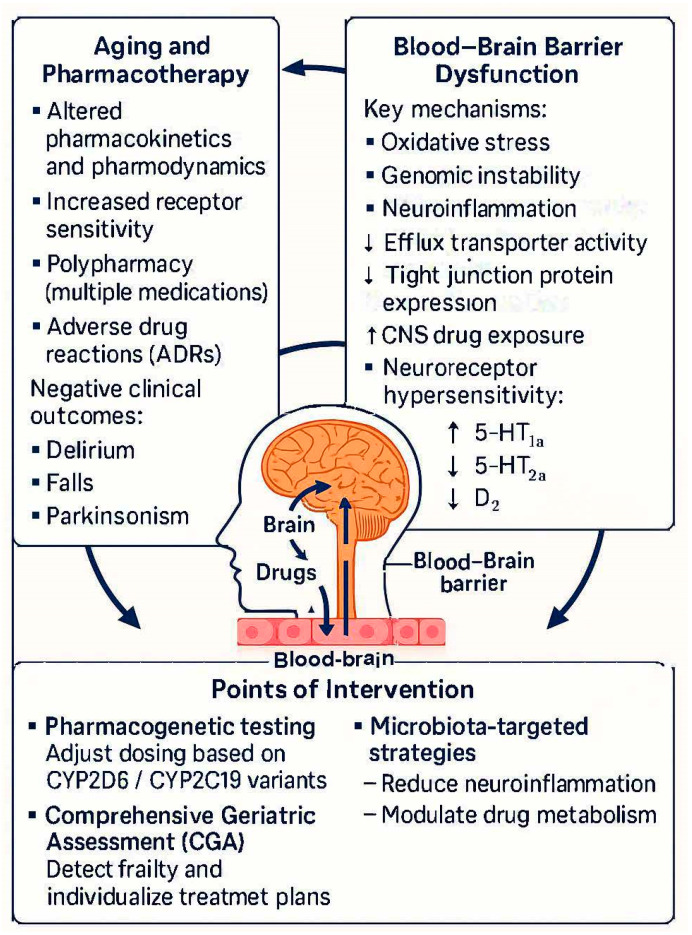
The blood–brain barrier in chronic pharmacotherapy in older adults. Created in Canva, Krita and other free graphic programs. ↓—decrease, ↑—increase.

The central nervous system (CNS) is protected by the highly selective blood–brain barrier (BBB), which precisely regulates the transport of substances between the blood and brain tissue. The integrity of this barrier is crucial for maintaining neuronal homeostasis and protecting against neurotoxins. In the aging process, and especially in the course of neurodegenerative diseases, the structure and function of the BBB undergo gradual degradation. This “leakiness” of the barrier has twofold pharmacological consequences: on one hand, it facilitates the penetration into the brain of drugs that normally do not cross it, increasing the risk of neurotoxicity, and on the other hand, it impairs the mechanisms for removing toxins and drugs from the CNS (Figure 2) [96,97,98,99].

### 3.1. The Neurovascular Unit: Structure and Age-Related Dysfunction

The modern understanding of the BBB goes beyond perceiving it as a simple layer of endothelial cells. It is a complex, dynamic structure, referred to as the neurovascular unit (NVU). In addition to specialized brain microvascular endothelial cells (BMECs), it includes pericytes, which surround the vessels and stabilize their structure, astrocyte end-feet, which are a type of glial cell, as well as microglia and neurons. The proper functioning of the BBB depends on close cooperation and communication between all these elements [100,101]. The aging process leads to pathological changes within the entire NVU. One of the key phenomena is the loss of pericytes, contractile cells that regulate blood flow in the microcirculation and are essential for maintaining barrier integrity. Their loss, observed both in the aging brain and in diseases like Huntington’s disease, leads to a weakening of the vessel structure and increased permeability [102,103].

Another characteristic phenomenon is reactive astrogliosis, the activation of astrocytes in response to injury or inflammation, which leads to glial proliferation [104]. Activated astrocytes can have both neuroprotective functions, by releasing growth factors, and neurotoxic functions, by producing pro-inflammatory cytokines and generating reactive oxygen species. In chronic inflammatory states, characteristic of aging (“inflammaging”), astrogliosis contributes to further barrier damage [105,106].

A fundamental structural element of the NVU is also the basement membrane, an extracellular matrix surrounding the vessels, composed of, among others, collagen and laminin proteins. Laminins are also part of the membrane surrounding the cell nucleus. It stabilizes the endothelial cells and is necessary for the formation of tight junctions. In aging and neurodegenerative diseases, due to excessive activity of matrix metalloproteinases (MMP), the basement membrane undergoes degradation, becoming thinner and discontinuous, which further weakens the barrier. These observations lead to an important conclusion: the “leakage” of the BBB in old age is not a simple mechanical damage but the result of a complex, progressive degradation of the entire neurovascular unit. It is underpinned by fundamental aging processes such as chronic, low-grade inflammation (“inflammaging”) and cellular senescence. Studies show that the accumulation of senescent endothelial cells and pericytes is directly related to impaired BBB integrity [107,108,109].

### 3.2. Molecular Integrity of the Barrier: Tight Junctions

The physical basis for the low paracellular (intercellular) permeability of the BBB are tight junctions (TJs). These are protein complexes that seal the space between adjacent endothelial cells, creating a continuous, impermeable barrier. The main molecular components of TJs are transmembrane proteins: claudins (especially claudin-5, crucial for the BBB), occludin and JAMs (Junctional Adhesion Molecules), which form the actual seal as ell as cytoplasmic proteins: proteins from the ZO family (Zonula Occludens: ZO-1, ZO-2, ZO-3), which link the transmembrane proteins to the actin cytoskeleton, stabilizing the entire structure [110,111,112,113,114].

The scientific literature presents seemingly contradictory data regarding the fate of these proteins in the aging process (Authors’ conclusion). On one hand, numerous studies, mainly based on immunohistochemical and imaging techniques, provide evidence for the disorganization and decrease in the amount of TJ proteins. For example, aged mice (24 months old) show significantly reduced expression of key TJ proteins—occludin and ZO-1—in brain capillaries, along with increased BBB permeability—IgG extravasation—compared to young mice (3 months) [115]. Similarly, in humans, aging and microvascular disease are associated with reduced expression of the occludin–ZO-1 complex and increased permeability across the BBB [116]. In the brains of aging mice and humans, as well as in models of neurodegenerative diseases, fragmented, weakened and discontinuous staining for claudin-5, occludin and ZO-1 is observed within the microcirculation. Studies on mouse models of accelerated aging (BubR1 hypomorphic) confirm a decrease in the levels of these proteins and impaired barrier integrity. These changes are often correlated with the loss of pericytes, suggesting that signals from pericytes are necessary to maintain the proper expression of TJ proteins by endothelial cells.

A stable mRNA level with a simultaneous decrease in the amount and disorganization of the protein is key to understanding the molecular mechanism of BBB damage. It indicates that the problem does not lie in the ability of aging endothelial cells to transcribe the relevant genes, but in post-transcriptional and post-translational processes [117,118,119]. This means that although the genetic material is correctly transcribed from DNA to mRNA, the cell fails at later stages: protein synthesis, its proper folding, modification (e.g., phosphorylation), transport to the cell membrane and incorporation into the tight junction structure [120]. Moreover, even correctly localized proteins may be excessively degraded, for example, by the aforementioned matrix metalloproteinases (MMPs), whose activity increases in inflammatory states [121].

### 3.3. The Role of P-Glycoprotein at the Blood–Brain Barrier

In addition to the physical barrier formed by TJs, the BBB has quite efficient active transport proteins that play a protective role for the brain. A key element of this system is P-gp, an efflux pump from the ABC protein family, which actively removes a broad spectrum of xenobiotics and potentially toxic metabolites back into the bloodstream. P-gp is responsible for removing not only drugs but also endogenous neurotoxins, such as beta-amyloid (Aβ) peptides [122,123].

Age- and sex-dependent changes: The function of P-gp at the BBB significantly weakens with age. This is confirmed by studies using positron emission tomography (PET) and the radiotracer [^11^C]-verapamil, which is a P-gp substrate. In older individuals, a larger Vd of this tracer is observed in the brain, which is inversely proportional to P-gp activity and indicates its reduced function [124]. Importantly, this decline is strongly sex-dependent. PET studies have shown that the age-related decline in P-gp function is a more rapidly progressing phenomenon mainly in men. In women, P-gp function remains relatively stable over the years. On one hand, the better-preserved P-gp function in older women may be a protective mechanism, limiting the accumulation of toxins in the brain later in life. On the other hand, the same studies showed that young women have a lower baseline P-gp function compared to young men. This may mean that throughout their lives, their brains are exposed to a greater cumulative exposure to neurotoxins, which theoretically could be one of the factors contributing to the higher incidence of Alzheimer’s disease in women [125].

Regulatory Mechanisms: The regulation of P-gp at the BBB is an extremely complex process that occurs at multiple levels. It includes the regulation of *ABCB1* gene transcription by transcription factors (e.g., NF-κB, PXR), post-transcriptional regulation by microRNAs, as well as numerous post-translational modifications of the protein itself, such as phosphorylation, glycosylation and ubiquitination, which directs the protein for degradation by proteasomes. Additionally, P-gp activity can be dynamically modulated by the local environment, e.g., by signaling factors such as VEGF or sphingolipids, which can cause “down-regulation” of proteins from the cell membrane to the cell interior, temporarily inactivating them [126,127,128,129,130].

## 4. Pharmacodynamic Changes: Receptors and Signaling Pathways

Pharmacodynamics describes what a drug “does” to the body—its interactions with molecular targets and the resulting biological response. In the aging process, significant pharmacodynamic changes occur, which mean that the same dose of a drug can produce a much stronger or weaker response in an older person than in a younger one. These changes result from modifications at the level of the receptors themselves, the signaling pathways that transmit the signal deep into the cell and the impairment of systemic homeostatic mechanisms [131].

### 4.1. General Principles of Altered Drug Sensitivity

The basic mechanisms underlying the altered response to drugs in the geriatric population include:

- Changes in receptor density: The number of receptors for a given neurotransmitter or drug on the cell surface may decrease (down-regulation) or increase (up-regulation) with age.
- Changes in receptor affinity: The binding strength of a drug to a receptor (Kd) may be modified.
- Changes in post-receptor response: Even with proper drug binding to the receptor, subsequent steps in the intracellular signaling cascade (e.g., production of second messengers, kinase activation) may be impaired.

Impairment of homeostatic mechanisms: With age, compensatory mechanisms that counteract excessive adverse drug effects in younger people weaken (e.g., reflex tachycardia in response to vasodilating drugs). These changes make geriatric patients particularly sensitive to the effects of drugs from many classes, which requires not only dose reduction but often the choice of alternative, safer therapies [132,133,134,135,136].

### 4.2. Quantitative Changes of Key Receptor Systems

Thanks to the development of molecular neuroimaging techniques, such as PET and single-photon emission computed tomography (SPECT), it has become possible to non-invasively, quantitatively study the density and availability of receptors in the living human brain. These studies have provided extensive data on how the aging process affects key neurotransmitter systems [137,138].

Adrenergic System: β-adrenergic receptors (βARs) play a key role in regulating the function of the cardiovascular and respiratory systems. Studies show that during aging, there is a decrease in the sensitivity of peripheral β-receptors. The vasodilatory response of blood vessels and the inotropic response of the heart to stimulation with β-adrenergic agonists decrease. This is supported, for example, by Tsujimoto et al., who reported a marked reduction in β-AR–mediated relaxation of rat mesenteric arteries with age [139]. This mechanism underlies, among other things, the increase in peripheral resistance and hypertension in old age. The decrease in sensitivity may result from a reduction in receptor density, their less efficient coupling with adenylyl cyclase or impaired cAMP generation. Interestingly, and highlighting the complexity of the changes, the opposite phenomenon is observed in the central nervous system—an increase in the density of β2 receptors in some brain areas, such as the hippocampus and frontal cortex. This tissue-specific, opposing regulation makes it difficult to predict the systemic effects of β-adrenergic drugs [140,141,142].

Dopaminergic System: Dopamine receptors, especially the D2 subtype, are a key therapeutic target for antipsychotic drugs and those used in the treatment of Parkinson’s disease. PET studies using radiotracers such as [^11^C]-raclopride generally indicate a linear decrease in the density of D2 receptors in the striatum with age [143]. However, this picture is complicated. There are two populations of D2 receptors: high-affinity and low-affinity for dopamine, with the high-affinity state considered active. Additionally, D2 receptor activity can show diurnal fluctuations, and the nature of these fluctuations may change with age—in younger people, a decrease in activity is observed in the evening, while in older people, an increase is observed [144].

Serotonergic System: The most comprehensive and quantitative data on the impact of aging on a neurotransmitter system come from systematic meta-analyses of PET and SPECT studies on the serotonergic system. The work of Karrer and colleagues, covering over 1000 healthy adults, has provided solid evidence for the differential aging rates of the individual components of this system [145]. 5-HT2A Receptors show the largest, strong decline in quantity with age, estimated at about 7% per decade. This decline is most pronounced in the cerebral cortex. 5-HT2A receptors are excitatory receptors involved in, among other things, learning and memory processes [146]. Serotonin transporter (SERT) shows a moderate decline in the number of available transporters, amounting to about 3% per decade. The largest changes are observed in the thalamus. SERT is the target of selective serotonin reuptake inhibitors (SSRIs) and is responsible for serotonin reuptake in the synapse [147].

Finally, 5-HT_1_A receptors are characterized by the slowest, slight decline in density, amounting to only 1.5% per decade. Moreover, presynaptic 5-HT_1_A autoreceptors, located in the raphe nuclei and regulating serotonin release, appear to be relatively preserved during aging. These observations are supported by neuroimaging meta-analyses of healthy adults. One drug acting on this receptor is buspirone (an anxiolytic) [148]. These differential rates of change for the individual elements of the serotonergic system lead to a fundamental conclusion: aging does not cause a significant inhibition of neurotransmission, but its modulation. The balance between individual neurotransmitters changes. The faster decline of excitatory 5-HT2A receptors with the relative preservation of inhibitory 5-HT1A receptors may underlie the age-related changes observed in the emotional and cognitive spheres, such as the preference for emotion-focused rather than problem-focused coping strategies [149,150,151]. These quantitative changes are summarized in Table 3. Moreover, the line between normal aging and “pathological” neurodegeneration seems to be blurring. The decline in serotonin transporter density, observed during aging, is also linked to the early stages of Alzheimer’s disease, and some data suggest it may be a driving factor of the pathology, not just its consequence [152]. PET studies in AD patients show a huge, up to 49%, loss of 5-HT1A receptors in the hippocampus, which is strongly correlated with the severity of cognitive deficits. This suggests that the physiological aging of the serotonergic system may reduce its functional reserve, making the brain more susceptible to neurodegenerative processes [153,154,155].

### 4.3. Clinical Consequences

Knowledge of molecular changes in receptors helps to understand the clinical phenomenon of hypersensitivity in geriatric patients to certain drugs. A classic example is drugs with anticholinergic effects. Many commonly used preparations—tricyclic antidepressants, first-generation antihistamines, drugs used in urology—have such properties [156]. In older adults, where there is a physiological decline in the activity of the cholinergic system, even small doses of these drugs can cause pronounced adverse effects. At the CNS level, this manifests as confusion, drowsiness, memory impairment and delirium [157] Peripherally, it leads to dry mouth, constipation, visual disturbances and urinary retention, which is particularly dangerous in men with benign prostatic hyperplasia. The increased sensitivity results from the fact that a drug blocking muscarinic receptors acts on a system that already has a diminished functional reserve [158,159,160,161].

## 5. Molecular Basis of Drug Interactions in Geriatrics

Drug–drug interactions (DDIs) are one of the greatest threats in geriatric pharmacotherapy. The risk of their occurrence increases with the number of drugs taken, and in patients using 10 or more preparations, it is almost certain. The consequences of DDIs can be diverse: from weakening or strengthening the therapeutic effect, through shortening or prolonging the duration of action, to the appearance of completely new, toxic effects. Understanding the molecular mechanisms underlying these interactions is key to predicting and avoiding them. In the geriatric population, these mechanisms are further potentiated by the previously discussed pharmacokinetic and pharmacodynamic changes [162,163,164].

### 5.1. Mechanisms of Pharmacokinetic Interactions

Pharmacokinetic interactions occur when one drug affects the LADME processes of another drug, changing its concentration in the body. In the context of a geriatric patient, the phenomenon of “reduced physiological reserve” is of key importance. The systems responsible for drug metabolism and elimination in older adults operate at a lower level of efficiency, making them much more susceptible to disturbances [165,166].

CYP450 Induction and Inhibition: The most common mechanism of DDIs is interaction at the level of cytochrome P450 enzymes. Competitive Inhibition: When two drugs are metabolized by the same CYP isoenzyme (e.g., CYP3A4 or CYP2D6), they compete for access to its active site. The drug with higher affinity (the inhibitor) slows down the metabolism of the other drug (the substrate), leading to an increase in its concentration and risk of toxicity. In a young person with full enzymatic activity, such inhibition may be partially compensated. In a geriatric patient, whose baseline CYP activity is already reduced by up to 30–40%, even partial inhibition can lead to a sharp drop in clearance and a dangerous increase in the concentration of the substrate drug. An example is the interaction between amiodarone (a CYP3A4 inhibitor) and statins (CYP3A4 substrates), which significantly increases the risk of myopathy [167,168,169]. Enzymatic Induction: Some drugs (inducers, e.g., rifampicin, carbamazepine) can increase the synthesis of CYP enzymes, accelerating the metabolism of other drugs. As mentioned earlier, the capacity for induction may be weakened in old age, making these interactions less predictable. Rifampicin is one of the main antibiotics used to treat tuberculosis. Carbamazepine is an anticonvulsant drug whose range of use can be much broader, e.g., treating trigeminal neuralgia [170,171,172].

Competition for Drug Transporters: A similar phenomenon of competition occurs at the level of transport proteins.

P-glycoprotein (P-gp) Inhibition: Many drugs can inhibit P-gp. Inhibition of P-gp in the intestine by an inhibitor drug (e.g., verapamil) can drastically increase the absorption and bioavailability of a substrate drug (e.g., digoxin, dabigatran), leading to its toxicity. In turn, inhibition of P-gp in the kidneys can block active tubular secretion. At the blood–brain barrier (BBB), inhibition of P-gp can increase central nervous system (CNS) exposure to drugs by reducing transporter-mediated efflux from the brain [173]. Drugs can also compete for uptake transporters in the liver (OATPs) and kidneys (OATs, OCTs), which affects their organ clearance [174].

### 5.2. Mechanisms of Pharmacodynamic Interactions

Pharmacodynamic interactions occur when drugs affect each other at the molecular target site (e.g., a receptor) or signaling pathway, without changing concentrations.

Synergism and Antagonism: Two drugs acting on the same receptor can have an additive effect (sum of effects), a synergistic effect (effect greater than the sum) or an antagonistic effect (effect less than expected). An example of synergism is the simultaneous use of drugs that depress the CNS (e.g., benzodiazepines and opioids), which significantly increases the risk of sedation and respiratory depression [175].

Additive Effect on Different Receptors: A classic example in geriatrics is the additive anticholinergic effect. Many patients simultaneously take several drugs from different groups (e.g., an antidepressant, a drug for overactive bladder, an antihistamine), each of which has some, even minor, cholinolytic effect. The summation of these effects leads to intensified symptoms of confusion, dry mouth or constipation, which is one of the most common problems in geriatric pharmacotherapy (see Table 4 for examples of clinically significant molecular interactions) [176].

## 6. The Role of Microbiome and Epigenetics

Traditional geriatric pharmacology has focused on changes in the physiology of the host itself. However, the latest research opens two new, fascinating fronts that are revolutionizing our understanding of individual variability in drug response during aging: the gut microbiome and epigenetics. These two areas show that pharmacokinetics and pharmacodynamics are not solely determined by a static genome and chronological age but are dynamically modulated by environmental factors and changes occurring throughout life [182,183].

### 6.1. The Gut Microbiome as a Regulator of Pharmacokinetics

The human gastrointestinal tract is inhabited by trillions of microorganisms, forming a complex ecosystem called the gut microbiota. There is growing evidence that this “second genome” plays a key role in health, disease and, importantly, in drug metabolism [184].

“Inflammaging” and Dysbiosis: In the aging process, the composition and function of the gut microbiota undergo significant changes. A decrease in its diversity, a reduction in the number of beneficial bacteria producing short-chain fatty acids (SCFAs), such as Faecalibacterium, and an increase in the number of opportunistic pathogens (pathobionts), especially from the Proteobacteria phylum, are observed. This imbalance, known as dysbiosis, contributes to the development of chronic, low-grade inflammation, referred to as “inflammaging,” which is one of the main drivers of aging processes and related diseases.

Molecular Mechanisms of Modulation: The gut microbiota can influence drug pharmacokinetics in several ways. First, the bacteria themselves can metabolize drugs, leading to their activation, inactivation or the formation of toxic products. Second, and perhaps more importantly, the microbiota can even regulate the expression of host genes, including genes encoding metabolic enzymes and drug transporters in the intestine and liver [182,183,184,185,186]. This mechanism is based on the production of metabolites by bacteria that act as signaling molecules. For example: Short-Chain Fatty Acids (SCFAs): Metabolites like butyrate, produced by fiber-fermenting bacteria, can influence the expression of transport proteins. Studies have shown that butyrate can increase the level of P-gp in intestinal cells [187].

Secondary Bile Acids: Bacteria modify primary bile acids produced by the liver, creating secondary bile acids. These, in turn, are ligands for the farnesoid X receptor (FXR) and the pregnane X receptor (PXR), which are key transcriptional regulators of many genes from the CYP and ABC families. Studies on germ-free mice colonized with specific bacterial strains have unequivocally confirmed this regulation. It has been shown that the presence of the gut microbiota can reduce the expression of P-gp (ABCB1) in the small intestine. In antibiotic-treated mice with depleted microbiota, an increase in P-gp expression was observed, resulting in reduced absorption and lower blood concentrations of tacrolimus, an immunosuppressive drug that is a P-gp substrate. This means that the composition of the microbiota can directly affect the bioavailability and effectiveness of treatment [182,183,184,185,186,187,188,189,190,191,192].

Therapeutic Potential: The dynamic and modifiable nature of the microbiome creates new therapeutic possibilities. Interventions such as the use of probiotics (live, beneficial bacterial strains), prebiotics (substances that stimulate the growth of beneficial bacteria, e.g., fiber) or targeted dietary modifications (e.g., the Mediterranean diet) can help restore the balance of the microbiota in older adults. Such actions could not only alleviate inflammation and improve general health but also potentially normalize the expression of transporters and enzymes, stabilizing drug pharmacokinetics and reducing its individual variability [189,190,191,192,193,194,195].

### 6.2. Pharmacoepigenetics: Epigenetic Regulation of Pharmacogenes in Aging

Pharmacogenetics, which studies the influence of inherited genetic variants on drug response, has revolutionized medicine, but it has a fundamental limitation: a patient’s genome is largely static throughout life. This does not explain why the response to the same drug in the same person changes with age. The answer to this question is provided by pharmacoepigenetics—a field that studies how reversible epigenetic modifications, which accumulate throughout life under the influence of environmental factors and the aging process itself, dynamically regulate the expression of genes related to pharmacokinetics and pharmacodynamics.

Epigenetics is the study of changes in gene expression that do not result from changes in the DNA sequence itself. The main epigenetic mechanisms are: DNA Methylation and histone modifications. The attachment of methyl groups to cytosines in DNA, usually in the promoter regions of genes. High promoter methylation generally leads to gene silencing (decreased expression). Histone proteins, around which DNA is wound, can undergo various modifications (e.g., acetylation, methylation). Histone acetylation usually “loosens” the chromatin structure, facilitating access for transcription factors and promoting gene expression, while some methylations “tighten” it, making it more condensed, which consequently silences specific genes [196,197,198,199,200,201]. There is growing evidence that the aging of the epigenome directly affects key genes involved in drug metabolism. Studies on animal models have shown complex, age-related changes in the methylation levels of genes encoding cytochrome P450 enzymes in the liver. For example, in aging mice, hypermethylation (increased methylation) and an associated decrease in the expression of the *Cyp2d9* gene (an ortholog of human *CYP2D6*) were observed. At the same time, the *Cyp1a2* gene underwent hypomethylation (decreased methylation), which paradoxically was also associated with a decrease in its expression. Human population studies have identified the *CYP2E1* gene as one of the main loci whose methylation changes in a manner highly correlated with age [202,203,204].

Changes in histone acetylation also play a key role. It has been shown that age-related changes in the level of acetylation of histone H3 at lysine position 9 in the regulatory regions of the CYP2E1 gene are correlated with its expression and metabolic activity in the liver. Similar relationships between histone acetylation and expression have been shown for the *Sult1a1* gene, which encodes a phase II enzyme—sulfotransferase [205,206].

The consistency and predictability of epigenetic changes in the aging process have led to the development of so-called “epigenetic clocks”. These are algorithms that, based on the level of methylation at several hundred specific sites in the genome, can estimate the biological age of an organism with high accuracy, which often differs from the chronological age. More importantly, an “acceleration” of the epigenetic clock (biological age higher than chronological age) is strongly associated with the risk of many diseases and mortality. There is strong evidence that such clocks may in the future become much better predictors of individual metabolic capacity and ADR risk than chronological age alone [207,208,209].

These discoveries have revolutionary scientific potential. First, they explain the dynamic nature of pharmacokinetic ageing. Second, since epigenetic modifications are reversible, they become attractive therapeutic targets. In the future, instead of merely adjusting drug doses to the ageing organism, it may become possible to use “epigenetic drugs” (e.g., histone deacetylase inhibitors, methylation modulators) to “rejuvenate” the epigenetic state of specific genes, restoring more youthful patterns of expression and function [210].

Looking ahead, the analytical advances discussed in this paper could be translated into clinical practice through a combination of targeted pharmacological and non-pharmacological interventions. In pharmacotherapy, pharmacoepigenetic and microbiota profiles could guide initial drug selection, individualize dosing and inform deprescribing decisions to minimize adverse drug reactions. For example, dose adjustments based on epigenetic markers or gut microbiota composition may optimize therapeutic efficacy while reducing toxicity. In parallel, lifestyle-oriented strategies—such as tailored nutrition plans, structured physical activity programmes and interventions to maintain social engagement—can support healthy ageing and enhance resilience to pharmacological stressors. Integrating these approaches into routine geriatric care would shift treatment paradigms from reactive symptom management toward proactive preservation of health and functional independence in older adults.

## 7. Clinical Implications

Translating fundamental knowledge about the molecular mechanisms of aging into daily clinical practice is a promising goal of geriatric pharmacology. Understanding how enzymes, transporters, barriers and receptors change opens the way for a more rational, safe and, most importantly, personalized approach to treating older adults. The era of “one dose fits all” medicine is coming to an end, and its place is being taken by strategies based on the individual patient’s profile (authors’ note).

### Pharmacogenomics in Geriatric Practice

Pharmacogenomics (PGx) is the field that studies how genetic variants in a patient’s genome affect their response to drugs. It is the most mature and best-validated branch of personalized medicine, which already offers concrete tools for optimizing therapy. For many drugs, there is strong evidence that polymorphisms in genes encoding metabolic enzymes or transporters are of key clinical importance. The best-known and clinically relevant drug–gene pairs include:

CYP2C19 and clopidogrel: clopidogrel is a prodrug activated by CYP2C19. Patients with loss-of-function variants (e.g., CYP2C19 *2/*2, *2/*3) exhibit reduced activation, leading to a weaker antiplatelet effect [211].

CYP2D6 and codeine/tramadol: these opioids require CYP2D6 to form active metabolites. Poor metabolizers (e.g., CYP2D6 *4/*4) have significantly weakened analgesic effects, while ultrarapid metabolizers risk opioid toxicity [212].

VKORC1 and CYP2C9 with warfarin: variants in VKORC1 (e.g., −1639G > A) and CYP2C9 (*2, *3 alleles) influence warfarin sensitivity and metabolism, enabling genotype-guided dosing to reduce bleeding risk [213]. Despite the availability of non-vitamin K oral anticoagulants (NOACs), vitamin K antagonists (VKAs) remain preferred in some cases, like mechanical heart valves.

Studies show that over 95% of individuals possess at least one actionable pharmacogenetic variant [214]. In the geriatric population, where polypharmacy is prevalent, the probability of a clinically significant drug–gene interaction is especially high. For instance, database analysis of 73 mln patients showed that in the group >65 years of age, approximately 50% of patients received at least one pharmacogenetic-dependent drug during the 4 years of observation [215]. Despite robust evidence and test availability, PGx integration into clinical practice remains limited by cost, infrastructure and clinician awareness. Overcoming these barriers is crucial to improving medication safety and outcomes in older adults [210,211,212,213,214,215,216,217,218,219,220,221].

## 8. Future of Geriatric Pharmacotherapy Development

Pharmacogenomics is a powerful tool, but it is only one piece of the puzzle. Truly personalized geriatric medicine must go beyond the genome and take into account the dynamic nature of the aging process (including modifications in gene expression).

The future of geriatric care lies in the synergistic use of various technologies:

Tests providing information about innate predispositions (pharmacogenetic), analysis of “epigenetic clocks” (e.g., DNA methylation) as a dynamic biomarker of biological age and risk indicator (epigenomics) and analysis of the gut microbiota composition to assess its impact on drug metabolism and inflammation (microbiomics) combined with traditional clinical data (results of a Comprehensive Geriatric Assessment (CGA), including an assessment of functional status, cognitive function, nutrition, frailty syndrome and social support) [222,223,224,225,226,227].

A look into the future of geriatric pharmacology reveals several promising directions. Therapies targeting the mechanisms of aging, the biggest breakthrough may come from the development of drugs that do not treat single diseases but target the fundamental processes of aging, i.e., senolytic drugs (which remove senescent cells), epigenetic modulators or interventions aimed at restoring balance in the metabolism of specific proteins may in the future form the basis for treating multimorbidity [228,229,230]. The progress may also stem from new clinical trial models. The traditional randomized controlled trial (RCT) model, focused on a single disease and excluding patients with multimorbidity, is inadequate for the geriatric population. It is necessary to develop new research methodologies that can assess the efficacy and safety of interventions in a group of patients with multiple conditions who are taking multiple drugs (authors’ conclusions).

As our knowledge of the role of epigenetics and the microbiome grows, and the technologies for their analysis become cheaper and more accessible, we can expect these biomarkers to be incorporated into routine diagnostics. Assessing the methylation profile of key pharmacogenes or analyzing the microbiota composition may become a standard element of personalizing therapy.

## Figures and Tables

**Table 1:** Changes in CYP activity in geriatric population [49,50,51,52,53].

Isoform	Age-Related Changes	Example Substrates	Clinical Information
CYP1A2	↓ Activity	Theophylline, caffeine, clozapine, imipramine	Increased theophylline concentration and risk of toxicity; intensified adverse effects of clozapine
CYP3A4	↓ Activity (by approx. 30–40%), ↓ First-pass effect	Statins (atorvastatin, simvastatin), calcium channel blockers (amlodipine, verapamil), benzodiazepines (alprazolam), macrolides, amiodarone	Increased drug concentration; risk of myopathy (statins), hypotension (calcium channel blockers), excessive sedation (benzodiazepines)
CYP2D6	↓ Activity (by approx. 20%)	Antidepressants (SSRIs, TCAs), antipsychotics (risperidone), β-blockers (metoprolol), opioids (codeine, tramadol)	Increased risk of adverse effects (e.g., serotonin syndrome, extrapyramidal symptoms); weakened analgesia (codeine, tramadol—prodrugs)
CYP2C19	↓ Activity	Proton pump inhibitors (omeprazole), clopidogrel, diazepam, antidepressants (citalopram)	Increased risk of ADRs; weakened effect of clopidogrel (prodrug), increased risk of cardiovascular events
CYP2C9	↓ Activity	NSAIDs (ibuprofen, diclofenac), warfarin, sulfonylurea derivatives	Increased risk of bleeding (warfarin, NSAIDs), hypoglycemia (sulfonylurea derivatives), toxicity (phenytoin)

↓—decrease.

**Table 2:** Changes in key drug transporters in geriatric population [87,88,89,90,91,92,93,94,95].

Transporter	Location	Age-Related Change	Example Substrates/Clinical Implications
OATP1B1 (SLCO1B1)	Liver (basolateral membrane)	↓ Expression/Function	Substrates: Statins (atorvastatin, pravastatin), methotrexate ↓ Hepatic uptake of drugs, ↑ plasma concentration, ↑ risk of myopathy (statins)
P-gp (ABCB1)	Intestine, liver, kidney, BBB	↓ Expression/Function	Substrates: Digoxin, dabigatran, anticancer drugs, loperamide; Inhibitors: Verapamil, amiodarone ↓ Drug elimination, ↑ CNS exposure, ↑ bioavailability of some drugs
BCRP (ABCG2)	Intestine, liver, kidney, BBB	↓ Expression/Function	Substrates: Statins (rosuvastatin), sulfasalazine, methotrexate, ↑ Statin concentration, ↑ risk of myopathy, altered pharmacokinetics
OAT1/OAT3 (SLC22A6/8)	Kidney	↓ Expression/Function	Substrates: NSAIDs, loop diuretics, penicillins, methotrexate ↓ Tubular secretion and renal clearance of anionic drugs, ↑ risk of toxicity
OCT2 (SLC22A2)	Kidney	↓ Expression/Function	Substrates: Metformin, cisplatin, procainamide ↓ Tubular secretion and renal clearance of cationic drugs, ↑ risk of lactic acidosis (metformin)

↓—decrease, ↑—increase.

**Table 3:** Quantitative changes in brain receptor density during aging (based on PET/SPECT studies) [137,138,139,140,141,142,143,144,145,146,147,148,149,150,151,152,153,154,155].

Receptor System	Molecular Target	Brain Region	Quantitative Change (Decline per Decade)
Serotonergic	5-HT2A Receptor	Cerebral cortex (globally)	7.0%
	5-HT Transporter (SERT)	Thalamus	3.0%
	5-HT1A Receptor	Parietal cortex	1.5%
Dopaminergic	D2-like Receptor	Striatum	approx. 2–8% (depending on study)
Adrenergic	β-adrenergic Receptor	Brain (cortex, hippocampus)	Increased density

**Table 4:** Examples of clinically significant drug interactions with a molecular basis in geriatrics [177,178,179,180,181].

Drug 1/Victim	Drug 2/Perpetrator	Molecular Mechanism	Potential Clinical Consequence in Geriatrics
Any drug with anticholinergic effects	Other drugs with anticholinergic effects (e.g., TCAs, drugs for overactive bladder, hydroxyzine)	Additive pharmacodynamic effect on muscarinic receptors	Intensified symptoms of confusion, delirium, dry mouth, constipation, urinary retention (additive anticholinergic burden)
Antidepressants (SSRIs)	Tramadol	Pharmacodynamic synergism (serotonergic effect)	Increased risk of serotonin syndrome, especially in patients with CYP2D6 polymorphism
Statins (simvastatin, atorvastatin)	Amiodarone, Verapamil	Inhibition of CYP3A4	Significant increase in statin concentration, increased risk of myopathy and rhabdomyolysis
Warfarin	Amiodarone, NSAIDs	Inhibition of CYP2C9 (amiodarone); displacement from albumin binding (NSAIDs)	Increase in concentration and free fraction of warfarin, sharp increase in INR, high risk of bleeding
Digoxin	Verapamil, Amiodarone	Inhibition of P-glycoprotein (P-gp) in the kidneys and intestines	Reduced renal clearance and increased absorption of digoxin, risk of toxicity (bradycardia, heart blocks)
Dabigatran	Verapamil, Dronedarone	Inhibition of P-glycoprotein (P-gp)	Significant increase in dabigatran concentration, high risk of serious bleeding

## Data Availability

Not applicable.
